# Network-based approach for targeting human kinases commonly associated with amyotrophic lateral sclerosis and cancer

**DOI:** 10.3389/fnmol.2022.1023286

**Published:** 2022-12-16

**Authors:** Fatima Khatoon, Shafiul Haque, Anwar Hashem, Ahmad Mahmoud, Hanaa Tashkandi, Darin Mathkor, Steve Harakeh, Badra Alghamdi, Vijay Kumar

**Affiliations:** ^1^Amity Institute of Neuropsychology and Neurosciences, Amity University, Noida, Uttar Pradesh, India; ^2^Research and Scientific Studies Unit, College of Nursing and Allied Health Sciences, Jazan University, Jazan, Saudi Arabia; ^3^Department of Medical Microbiology and Parasitology, Faculty of Medicine, King Abdulaziz University, Jeddah, Saudi Arabia; ^4^Vaccines and Immunotherapy Unit, King Fahd Medical Research Center, King Abdulaziz University, Jeddah, Saudi Arabia; ^5^College of Applied Medical Sciences, Taibah University, Medina, Saudi Arabia; ^6^Department of General Surgery, Faculty of Medicine, King Abdulaziz University, Jeddah, Saudi Arabia; ^7^King Fahd Medical Research Center, and Yousef Abdullatif Jameel Chair of Prophetic Medicine Application, Faculty of Medicine, King Abdulaziz University, Jeddah, Saudi Arabia; ^8^Department of Physiology, Neuroscience Unit, Faculty of Medicine, King Abdulaziz University, Jeddah, Saudi Arabia

**Keywords:** kinases, ALS, cancer, network biology, miRNAs, drugs

## Abstract

**Background:**

Amyotrophic Lateral Sclerosis (ALS) is a rare progressive and chronic motor neuron degenerative disease for which at present no cure is available. In recent years, multiple genes encode kinases and other causative agents for ALS have been identified. Kinases are enzymes that show pleiotropic nature and regulate different signal transduction processes and pathways. The dysregulation of kinase activity results in dramatic changes in processes and causes many other human diseases including cancers.

**Methods:**

In this study, we have adopted a network-based system biology approach to investigate the kinase-based molecular interplay between ALS and other human disorders. A list of 62 ALS-associated-kinases was first identified and then we identified the disease associated with them by scanning multiple disease-gene interaction databases to understand the link between the ALS-associated kinases and other disorders.

**Results:**

An interaction network with 36 kinases and 381 different disorders associated with them was prepared, which represents the complexity and the comorbidity associated with the kinases. Further, we have identified 5 miRNAs targeting the majority of the kinases in the disease-causing network. The gene ontology and pathways enrichment analysis of those miRNAs were performed to understand their biological and molecular functions along with to identify the important pathways. We also identified 3 drug molecules that can perturb the disease-causing network by drug repurposing.

**Conclusion:**

This network-based study presented hereby contributes to a better knowledge of the molecular underpinning of comorbidities associated with the kinases associated with the ALS disease and provides the potential therapeutic targets to disrupt the highly complex disease-causing network.

## Introduction

Amyotrophic lateral sclerosis (ALS) is a fatal neurodegenerative disease, characterized by the progressive degeneration of upper and lower motor neurons in the brain, in the brainstem, and in the spinal region. This neuronal degeneration leads to progressive skeletal muscle atrophy and death, by respiration failure within 2–5 years from the onset of symptoms (Alonso et al., 2009). ALS is a heterogeneous disease where several pathophysiological processes have been demonstrated to induce neuronal death, including oxidative stress, mitochondria impairment, growth factor deficiency, neuro-inflammation, defective axonal transport, RNA metabolism, aberrant stimulation of kinase activity, impaired brain energy metabolism, autophagy, and stress-induced cell death (Taylor et al., 2016). Recent studies also reported several other causative genes that encode for kinases and are involved in ALS and other neurodegenerative diseases (Guo et al., 2020).

Kinases are enzymes that function as transferases to catalyze almost every signal transduction process and pathway by adding a phosphate group (PO_4_^3−^) to hydroxyl groups of substrates such as amino acids, nucleic acids, as well as lipids (Rask-Andersen et al., 2014). Based on their substrate binding, kinases are classified into protein, lipid, and nucleotide kinases. Kinases are involved in several biochemical reactions associated with proteins, lipids, and nucleotides metabolism (Higelin et al., 2018). The phosphorylation of protein *via* kinases stimulates the majority of the cell life processes, while the abnormal phosphorylation leads to the consequences of diseases, such as human cancer initiation and progression. Apart from the oncological issues, disruptive kinase activity has been demonstrated in several other human diseases such as immune, neurological, and infectious diseases (Bhullar et al., 2018). Thus, the discovery of kinases provides clarity to understand the cellular pathways, disease mechanisms and to develop their therapeutic drugs.

In this study, we have used a network-based system biology approach to investigate the kinase-based molecular interplay between ALS and other human disorders. To date, multiple network-based analysis has been reported to identify the target genes in the network (Prasad et al., 2020, 2021a,b,c,d). Here, firstly we retrieved 62 ALS-associated kinases from several recent studies (Guo et al., 2020; García-García et al., 2021; Palomo et al., 2021; Sahana and Zhang, 2021) and databases including ALSoD (Abel et al., 2012) and Malacard (Rappaport et al., 2017). The protein–protein interaction (PPI) network of the identified 62 kinases was prepared to understand the association between these kinases. Further using these kinases, we identified their associated diseases by scanning multiple disease-gene interaction databases to understand the link between the ALS-associated kinases and other diseases. A disease-kinase interaction network was prepared to have 36 kinases associated with 381 different diseases and make a total of 603 disease-kinase interactions, which ultimately indicates the complexity and comorbidities associated with the ALS-linked kinases. Next, we explore the miRNAs as a potential therapeutic agent against the identified disease-causing kinases. We have prepared a miRNA-kinase interaction network and identified the top 5 miRNAs having interactions with the majority of the kinases in the network suggesting a potential therapeutic target. Similarly, we have screened multiple drug-gene interaction databases to identify drug molecules interacting with the kinases and finally identified 3 drug molecules having interactions with the majority of kinases in the network. This study will thus lead to the identification of potential drug candidates for disrupting the disease-causing network related to the ALS-associated-kinases.

## Materials and methods

### Data collection

The list of Genes associated with ALS was retrieved from ALSoD (Abel et al., 2012) and Malacard (Rappaport et al., 2017) databases. The list of human kinases was retrieved from the kinome database (Manning et al., 2002). Next, those kinases that were associated with the ALS, reported in ALSoD and Malacard databases were used in the study. Apart from these two databases, we have also retrieved recently reported kinases involved in ALS from many studies including Guo et al. (2020), García-García et al. (2021), Sahana and Zhang (2021), and Palomo et al. (2021).

### Protein–protein and disease-gene interaction study of the selected kinases

After obtaining the list of kinases associated with ALS, we prepared a PPI network of the kinases to identify how well connected these kinases are with each other. Further, the kinases showing high-density interaction with each other were used to scan the DisGeNET database (Piñero et al., 2017) to find out the involvement of the kinases in other diseases including cancer. The DisGeNET database includes the information of human variant-disease associations and gene-disease associations from several repositories such as environmental, complex, and Mendelian diseases. The retrieved information was used to prepare the disease-gene interaction network by using the Cytoscape tool (Shannon et al., 2003).

### Identification of hub gene among the kinases involved in ALS and other diseases

Hub genes in the network are those genes that have the highest number of direct interactions with other nodes in a network. For identifying the hub genes in a network, a PPI network of the kinases involved in ALS and other diseases was prepared using the STRING plugin of the Cytoscape tool (Shannon et al., 2003). The STRING plugin includes direct and indirect association with gene fusion, text-mining, co-expression, neighborhood, and experimental data for preparing the PPI network. The network analyzer tool of the Cytoscape tool was used to calculate the topological properties of the network such as degree of connectivity and betweenness centrality values. Nodes having a higher number of degrees of connectivity and betweenness centrality score were considered as hub genes in the network. Briefly, degree (k) signifies the number of interactions made by nodes in a network, and is expressed as:

Degree centrality (k) = $\underset{a\varepsilon K_{b}}{\sum }w\left(a,b\right).$

Where, $K_{a}$ is the node-set containing all the neighbors of node u, and *w (a, b)* is the edge weight connecting node *a* with node *b*.

Betweenness centrality ( $C_{b}$ ) represents the degree to which nodes stand between each other based on the shortest paths. A node with higher betweenness centrality represents more control over the network. It is expressed as:

$C_{b}$ (u) = $\underset{k\neq u\neq f}{\sum }\frac{p\left(k,u,f\right)}{p\left(k,f\right)}$

Where *p (k, u, f)* is the number of interactions from *k* to *f* that passes through *u*, and *p (k, f)* denotes the total number of shortest interactions between node *k* and *f*.

### Gene ontology and pathway enrichment study

For a comprehensive analysis of the biological functions of kinases, we have used the Enrichr web server (Kuleshov et al., 2016) to study the functional enrichment of the kinases. The Enrichr is an integrative web-based software application that includes new gene set libraries for analyzing gene sets generated by genome-wide experiments. The gene ontology analysis included the annotation at the biological level, cellular level, and molecular level. Kyoto encyclopedia of genes and genomes (KEGG) database was used for the pathways enrichment analysis of the concerned kinases. The pathways and functions with *p* < 0.05 were considered significantly enriched.

### miRNA-gene interaction analysis

Further, the microRNAs (miRNAs) were identified as a potential drug target against the selected kinases, and the interacting miRNAs were screened out from the miRNet database (Fan and Xia, 2018). The miRNet database integrates four well-annotated databases including miRTarBase v8.0, TarBase v8.0, and miRecords, and provides miRNA interaction with several hosts organism including humans. The miRNA-Gene interaction network was created using the Cytoscape tool (Shannon et al., 2003).

### Drug-gene interaction analysis

Apart from miRNAs, the drug targets were also identified against the selected kinases by screening DrugBank (Wishart et al., 2006) and DGIdb databases (Cotto et al., 2018). DrugBank is a unique bioinformatics/chemoinformatics resource that combines detailed drug data with comprehensive drug target information. DGIdb is an open-access database and web interface with open-source code The predicted drugs were used to construct the drug-gene interaction network by using the Cytoscape tool.

### Survival analysis of hub kinases from TCGA database

The correlation of hub kinases expression and overall survival from upregulated and downregulated PPI networks was accessed using the UALCAN database (Chandrashekar et al., 2017). UALCAN database is an interactive web portal that provides the survival analysis of TCGA data by using Kalpen-Meier analysis. The Kalpen Meier analysis determines the survival rate and hazard using expression and available clinical data of the patients. Two expression groups, i.e., high expression and low expression were defined using median kinase value as a cutoff threshold. Analysis having a value of *p* < 0.1 was considered statistically significant.

## Results

### Identification of ALS specific human kinases

For identifying the kinases specific to ALS disease, several kinase databases, and disease-gene interaction databases were screened. A list of 650 human kinases was retrieved from the Kinome database, whereas a list of 474 genes reported with ALS diseases was retrieved from the MalaCard database. Out of these 650 and 474 genes, a total of 21 ALS-associated kinases were identified and further used in the study. Apart from databases, several literatures were also screened to identify the ALS-associated kinases (Chang et al., 2020; García-García et al., 2021; Palomo et al., 2021; Sahana and Zhang, 2021). A total of 41 kinases associated with the ALS disease were identified from the literature search. Finally, a list of 62 (21 + 41) kinases was prepared (Supplementary Table S1). Further, a PPI network between the selected kinases was prepared using the STRING plugin of the Cytoscape tool to identify the interaction among the kinases. Out of 62 kinases, 56 kinases were preparing the interaction network (Supplementary Figure S1). A network having 56 nodes and 196 protein–protein interactions was prepared. Those 56 kinases were further used in the study.

### Generation of disease-kinase and kinase-kinase interaction network specific to ALS-associated kinases

**Figure 1:**
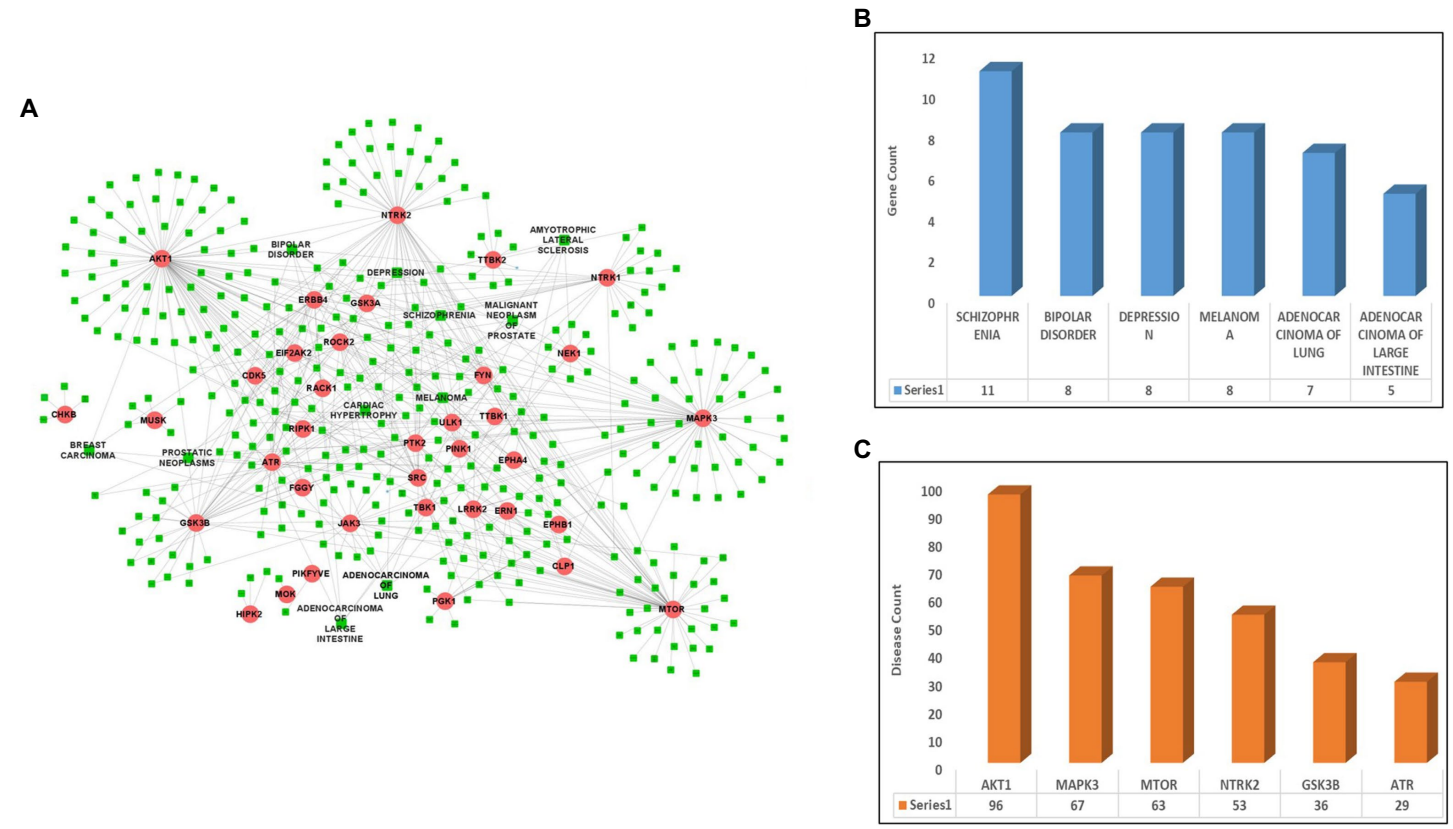
Disease-kinase interaction network. **(A)** ALS-associated 36 Kinases (Red) show interaction with the human diseases along with ALS (Green). **(B)** Bar plot representing the highest number of genes associated with the disease. **(C)** Bar plot representing the highest number of diseases associated with the genes.

After identifying the list of 56 ALS-associated highly interacting human kinases, the DisGeNet database was scanned to identify the link between the selected kinases and other diseases. After screening the database, a total of 36 kinases were identified showing their role in other diseases such as lung cancer, breast cancer, melanoma, and others along with ALS. A disease-kinase interaction network was prepared using the Cytoscape tool having 36 kinases associated with 381 different diseases and making a total of 603 disease-kinase interactions (Figure 1A; Supplementary Table S2). The disease-kinases interaction network showed that several disorders were connected with more than one kinase in the network such as schizophrenia (*n* = 11), bipolar-disorder (*n* = 8), depression (*n* = 8), melanoma (*n* = 8), adenocarcinoma of lungs (*n* = 7) and adenocarcinoma of large intestine (*n* = 5; Figure 1B).

Similarly, the disease-kinase interaction network also reveals that many of the disorders share a common genotype. For example, AKT1 (*n* = 96), MAPK3 (*n* = 67), mTOR (*n* = 63), NTRK2 (*n* = 53), GSK3B (*n* = 36) and ATR (*n* = 29) kinases are linked to the multiple disorders (Figure 1C). These molecular overlapping represents a highly-clustered-high-density disease network and suggests patients having altered forms of ALS-associated kinases are more prone to the other diseases.

**Figure 2:**
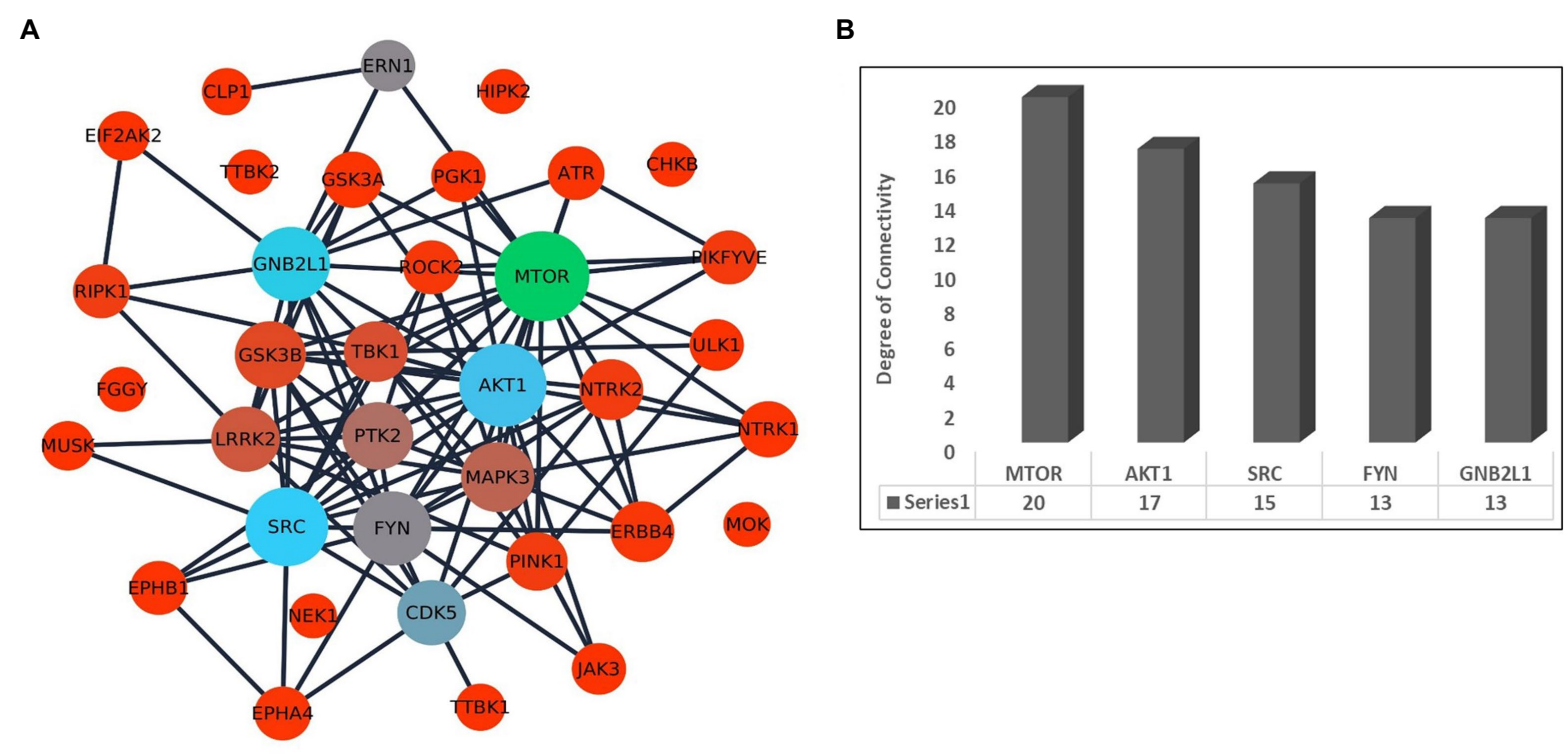
**(A)** Interaction network of kinases associated with ALS and other diseases in humans. The size of the node depends on its degree of connectivity. **(B)** Bar plot showing the degree of connectivity value of top 5 hub genes.

Further, we have prepared a PPI network to identify the association between the kinases. The selected 36 kinases show very high interaction among each other. The network topological properties such as degree of connectivity and the betweenness centrality values of the nodes in the network were calculated using the network analyzer tool to identify the hub genes in the network. The hub genes in the network are those genes that are highly connected with the other nodes in the network on a direct basis (Figure 2A). Any change in the expression of the hub genes in the network can influence the major part of the network. It is also suggested to target the hub genes in the network to disrupt the disease-causing network. The top hub genes in the PPI network are MTOR (*k* = 20), AKT1 (*k* = 17), SRC (*k* = 15), FYN (*k* = 13), and GNB2L1 (*k* = 13; Figure 2B; Supplementary Table S3).

### Gene ontology and pathway enrichment analysis of selected kinases

**Figure 3:**
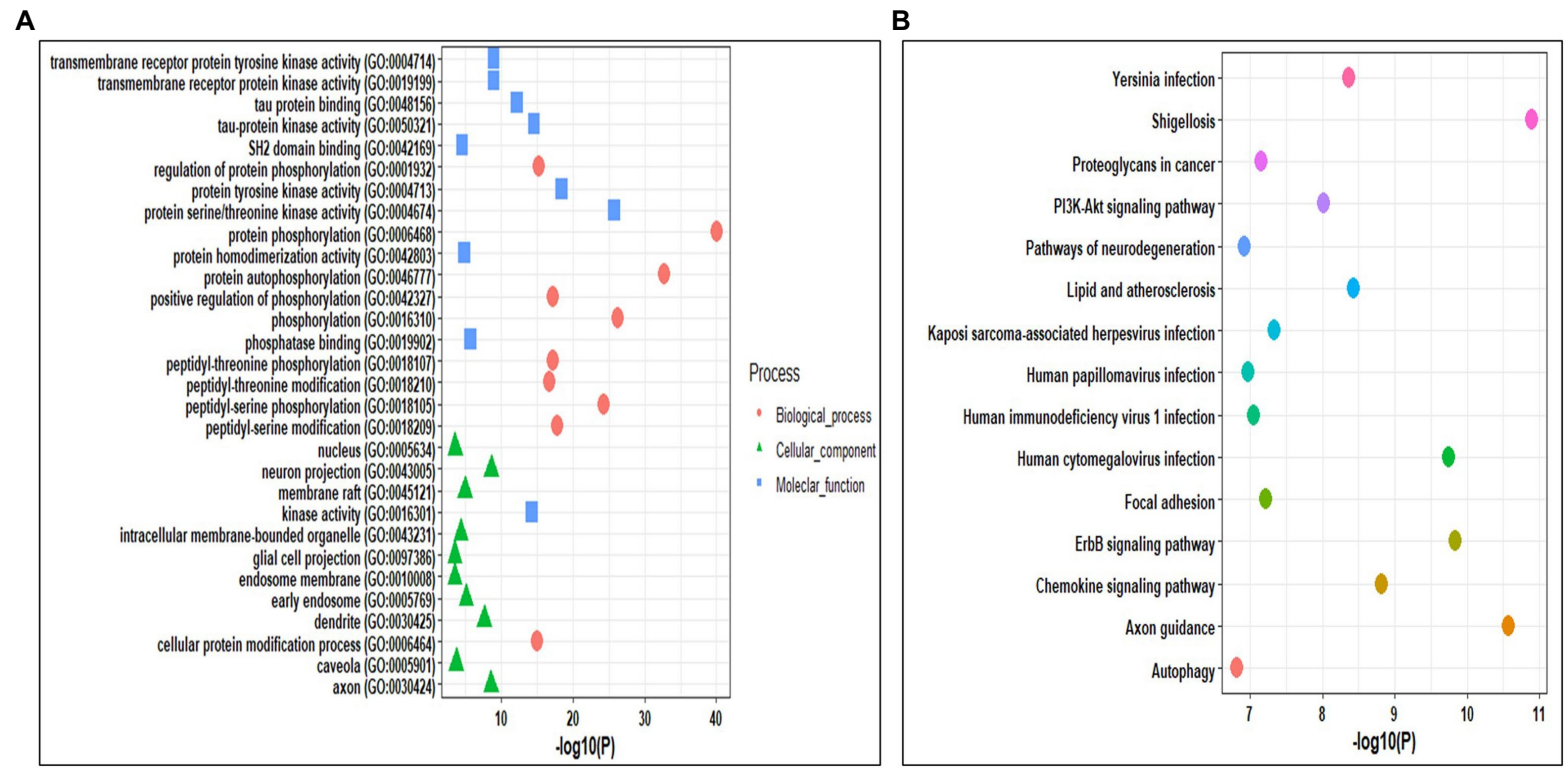
**(A)** Gene Ontology enrichment analysis of the identified kinases. **(B)** KEGG pathway enrichment analysis of the identified kinases.

Gene ontology analysis helps in identifying the roles of the genes at biological, cellular, and molecular levels. The Gene ontology analysis of kinases was performed using the Enrichr tool. The selected kinases were mainly enriched in biological processes such as protein phosphorylation and auto-phosphorylation, peptidyl-serine and peptidyl-threonine phosphorylation and modification, regulation of protein phosphorylation, and cellular protein modification process. In cellular components, the kinases were mainly enriched in neuron projection, axon, dendrite, early endosome, membrane raft, intercellular membrane-bounded organelle, caveola, nucleus, endosome membrane, and glial cell projection. Whereas, the molecular functions were enriched in kinase activity, protein serine/threonine kinase activity, protein tyrosine kinase activity, tau-protein kinase activity, and binding, transmembrane receptor protein kinase activity, transmembrane receptor protein tyrosine kinase activity, phosphatase binding, protein homodimerization activity, and SH2 domain binding (Figure 3A; Supplementary Table S4). We also performed the pathways enrichment analysis of kinases using KEGG from the Enrichr webserver. The kinases were mainly enriched in axon guidance, ErbB signaling pathways, human cytomegalovirus infection, chemokine signaling pathway, lipid and atherosclerosis, yersinia infection, PI3K-Akt signaling pathway, and focal adhesion pathways (Figure 3B; Supplementary Table S4).

### Identification of miRNA and drug molecules as a potential therapeutic target

**Figure 4:**
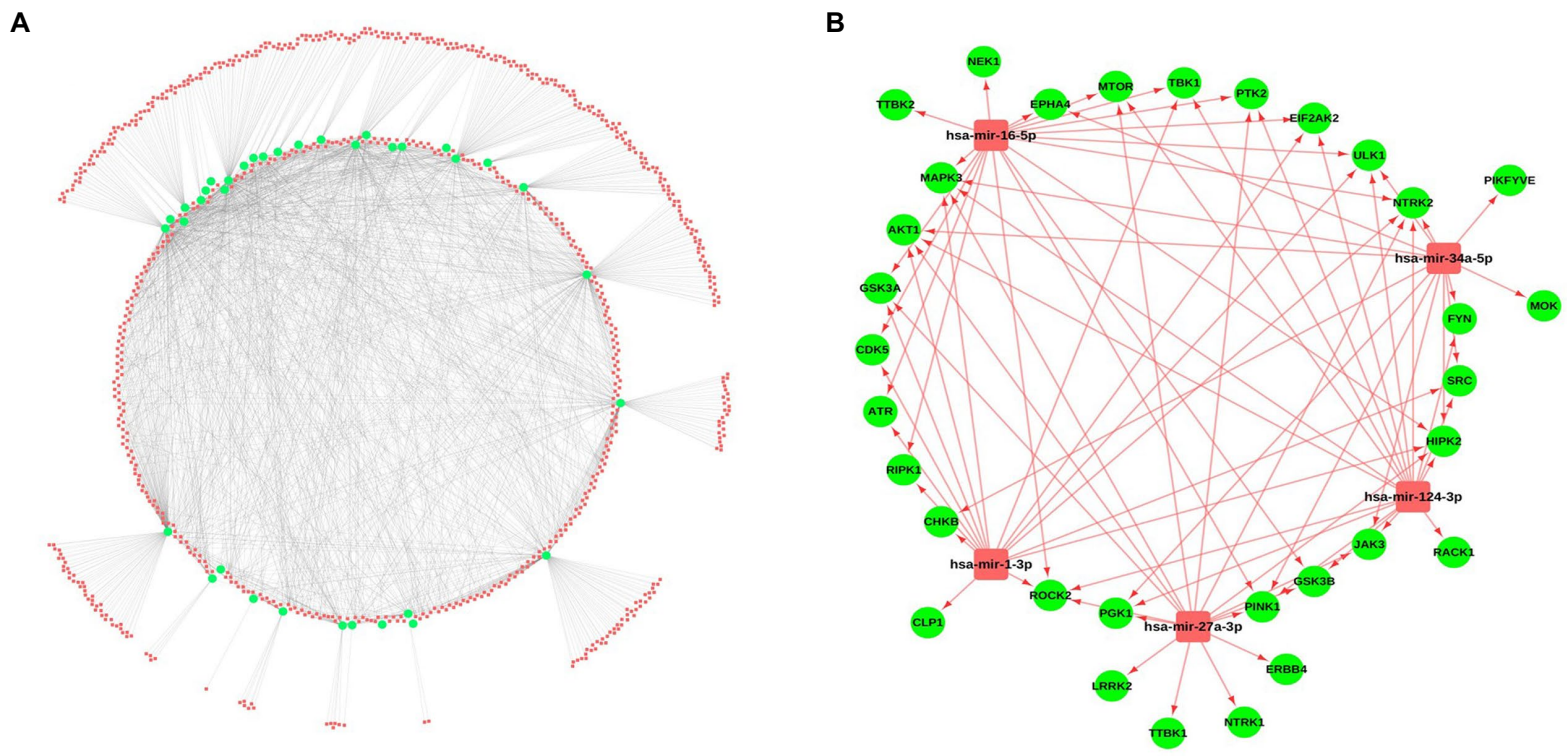
miRNA-kinases interaction network. **(A)** The kinases (Green) show interaction with the miRNAs from several databases including miRNet, miRTarBase, and TarBase. **(B)** Interaction of the top 5 miRNAs, selected based on a high degree of connectivity with 29 kinases.

Micro-RNAs are the small non-coding RNAs, involved in the expression of genes by interacting with mRNAs. For identifying the miRNAs interacting with the selected kinases, several miRNA-gene interaction databases such as miRNet and miRTarBase were screened. A total of 788 miRNAs were identified showing the interaction with the selected kinases. A miRNA-kinase interaction network having 788 miRNAs, 36 kinases, and 1891 miRNA-kinase interaction was prepared using the Cytoscape tool (Figure 4A; Supplementary Table S5). Further, we have calculated the topological properties of the miRNA-kinase network and selected the top 5 highly interactive miRNAs in the network based on their degree value. These top 5 miRNAs were interacting with 29 kinases out of 36 kinases, suggesting that these miRNAs can be potentially used as a therapeutic target. Out of 5 miRNAs, has-miR-16-5p hsa-miR-124-3p were showing the highest interaction with a maximum 17 number of kinases, hsa-miR-27a-3p, hsa-miR-1-3p and hsa-miR-34a-5p were showing interaction with 16, 15, and 14 kinases, respectively (Figure 4B).

**Figure 5:**
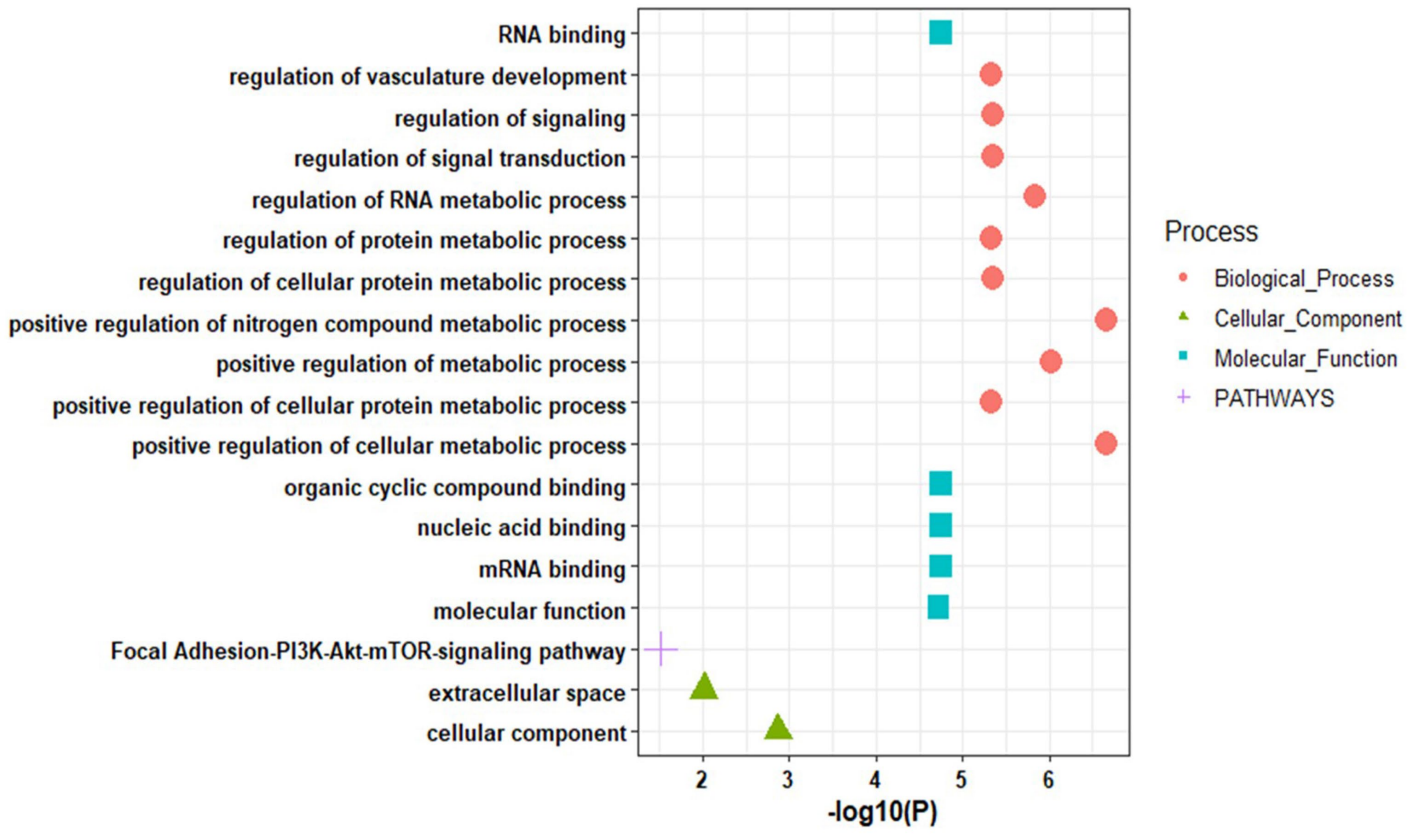
Gene ontology of top 5 miRNAs interacting with kinases.

Further, the enrichment analysis of top identified miRNAs reveals their role in biological processes such as positive regulation of the cellular metabolic process, regulation of RNA metabolic process, regulation of signal transduction, regulation of signaling, regulation of vasculature development, and others. In molecular functions, the miRNAs are mainly enriched in RNA binding, mRNA binding, nucleic acid binding, and organic cyclic compound binding. These miRNAs showed enrichment in the focal adhesion-PI3K-Akt–mTOR-signaling pathway (Figure 5; Supplementary Table S6).

**Figure 6:**
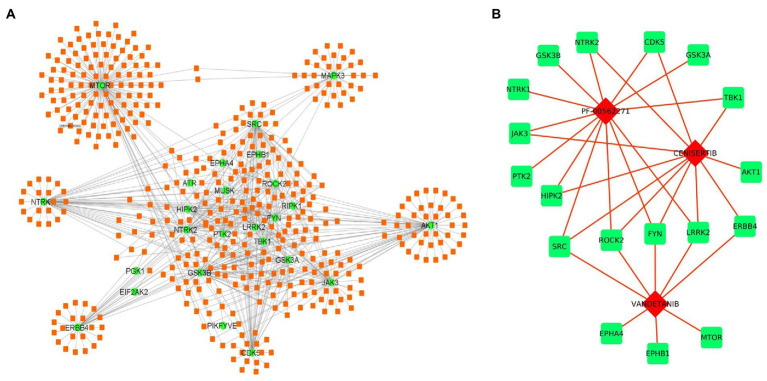
Drug-kinase interaction network. **(A)** The 36 kinases (Green) showed interaction with 468 drugs (Orange) from the DGI database. **(B)** Drug-kinase interaction of the top 3 selected drugs having association with the majority of kinases in the network.

Apart from miRNAs, we have also identified the potential drug target against the selected kinases. Several drug-gene interaction databases were screened to identify the drugs interacting with selected kinases. We have identified a total of 467 drug molecules showing interactions with 36 kinases (Figure 6A; Supplementary Table S7). Further based on interactions, we have identified 3 drugs molecules namely, PF-00562271, Cenisertib, and Vandetanib interacting with 13, 11, and 8 kinases, respectively. The result thus suggests the therapeutic potential of the identified drugs either in an individual manner or in combination as well (Figure 6B).

### Survival analysis of hub kinases

**Figure 7:**
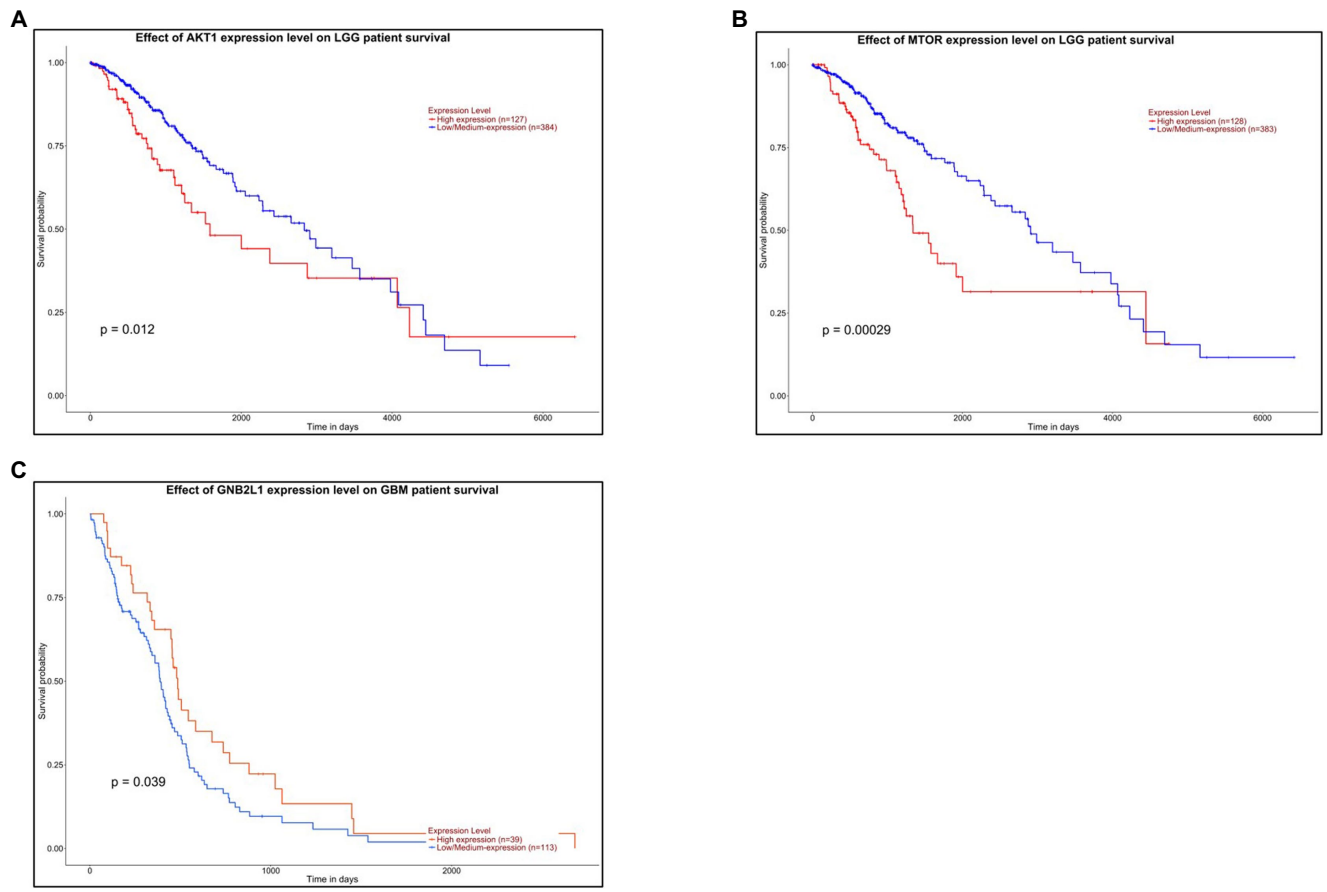
Survival Analysis. The Kalpen Meier survival analysis of **(A)** AKT1 and **(B)** MTOR gene in LGG cancer patients. **(C)** The Kalpen Meier survival analysis of GNB2L1 gene in GBM cancer patients.

Survival analysis of hub kinases from upregulated and downregulated PPI networks was performed by Kalpen Meier analysis using the UALCAN web portal. A threshold of value of *p* < 0.1 was applied to identify a statistically significant prognostic marker. Out of the 5 hub kinases namely AKT1, GNB2L1, FYN, SRC, and mTOR from the PPI network, only 2 kinases namely AKT1 and mTOR in LGG, and only one kinase GNB2L1 in GBM had a value of *p* < 0.1 and was considered a probable potential biomarker for prognosis. The survival analysis chart of the AKT1 gene in LGG cancer patients reveals that patients had higher expression of the AKT1 gene and had more survival probability and living time (in days) as compared to patients who had lower AKT1 gene expression (Figure 7A). However, in LGG cancer patients, the lower expression of the mTOR gene represents a higher survival rate in patients (Figure 7B). Whereas, in GBM cancer patients, the higher expression of the GNB2L1 gene represents a higher rate of survival in patients as compared to patients having a lower expression of the GNB2L1 gene (Figure 7C).

## Discussion

Kinases are the dynamically signaling proteins that act as a switch in the cell by phosphorylating target proteins. Several studies have been reported that the abruption in kinases activity or disturbances in the kinome network may cause various neurodegenerative diseases and cancers in humans (Freedman et al., 2013). In this study, we have adopted a network-based system biology approach to investigate the kinase-based molecular interplay between ALS and other human disorders.

Here, we analyzed the disease-kinase network of ALS-associated kinases that resulted in a disproportionately large number of disease-kinase associations. It includes diseases such as schizophrenia, bipolar disorder, depression, melanoma, cardiac diseases, adenocarcinoma of the lung, adenocarcinoma of the large intestine, malignant neoplasm of prostate, and prostatic neoplasms, which represents the higher connection with kinases.

### Kinases commonly involved in ALS and cancers

The significant finding of this study is the identification of 28 kinases that are commonly linked to ALS as well as various type of human cancers. Among the common kinases, the mTOR, AKT1, SRC, FYN, and GNB2L1 are the kinases that are common in ALS and cancers and also identified as hub genes from the PPI network. The mechanistic/mammalian target of rapamycin (mTOR) is a Serine/Threonine kinase, that plays a central role in regulating human physiological activities including tissue regeneration, regulatory T cell differentiation, and function, and various types of cancers (Sabatini, 2017). The interruption of mTOR signaling results in several disorders including cancers, diabetes, obesity, and neurodegenerative diseases (Huang, 2020). In certain human diseases, it is considered promising to target mTOR pathways according to their physiological role. Another hub gene, the AKT1 is a serine/threonine kinase, involved in the stimulation of several cellular functions, including cell proliferation, migration, growth, and cell survival. It also play important role in the initiation of protein synthesis, cell metabolism, and immune cell activity (Henderson et al., 2015). It is reported that it influences all aspects of cancer biology and has clinical relevance to the outcome of cancer therapy (Szymonowicz et al., 2018). Whereas, a decrease in AKT1 activity is associated with ALS (Wang et al., 2019). Another hub kinase RACK1 (GNB2L1), is a highly conserved intracellular adaptor protein and involved in several biological processes including virus infection, cell migration, neural development, and angiogenesis. It also functions as an anchoring protein for the activation of protein kinase C (PKC; Li and Xie, 2015). The SRC family of protein tyrosine kinases (SFKs) is one of the kinases associated with ALS and cancer. It plays a central role in the activation of signal transduction *via* an extensive set of cell surface receptors in the context of several cellular environments. The SFKs are also involved in several cellular processes such as cell growth, shape, differentiation, migration, specialized cell signals, and survival (Parsons and Parsons, 2004). The FYN kinase is also involved in neurodegenerative diseases and cancers. It belongs to the SRC family and plays important role in several signal transduction pathways such as axon guidance, myelination, synaptic transmission, and oligodendrocyte formation in the central nervous system (Matrone et al., 2020). Recent studies report its role in molecular signaling pathways underlying neurodevelopment as well as neuropathological events (Matrone et al., 2020).

In addition, the other identified common kinases in ALS and human cancers, including the homeodomain-interacting protein kinase 2 (HIPK2), is a serine–threonine kinase, that participates in the regulation of gene expression, signal transduction, and apoptosis regulations. It is well known for its pathological role in human cancers. Whereas, the HIPK2 has also been reported in neurodegenerative diseases *via* endoplasmic reticulum (ER) stress (Feng et al., 2017). Further, the cyclin-dependent kinase (CDK5) is a serine/threonine kinase, belongs to the mitotic cyclic-dependent kinases family. It is characterized by its role in the central system for axon elongation, neuronal migration, and differentiation rather than in the cell cycle (Pozo and Bibb, 2016). It is also involved in the microtubular arrangement, sorting of axodendritic cargos, (Klinman et al., 2017) and phosphorylation of NF-H subunit to stimulate axonal transport in neurons (Shea et al., 2004). The CDK5 has been reported in the development of various types of human cancers including breast, colon, lung, pancreatic, and brain tumors (Pozo and Bibb, 2016). In addition, we also identified EPHA4 as a common kinase in ALS and cancers. EPHA4 is a tyrosine kinase that belongs to the Ephrin receptor subfamily. It stimulates axonal guidance in the corticospinal tract, and also functions as a mediator of inflammation in spinal cord injury (Goldshmit et al., 2004; Zhao et al., 2018). The EPHA4 influences motor neuron degeneration and disease progression in ALS (Van Hoecke et al., 2012), while, in another study, it is reported that downregulation of EPHA4 signaling enhances the functionality and motor neuronal survival (Zhao et al., 2018). Even though these results indicate that EPHA4 receptor tyrosine kinase may serve as a therapeutic target for ALS. Another kinase, the ERBB4 that encodes Erb-B4 receptor tyrosine kinase 4, is involved in important cellular processes, including neurodevelopment (Takahashi et al., 2013). It also activates multiple signal transduction proteins such as mTORC1, mitogen-activated protein kinase (MAPK), STAT, and Agrin/MuSK pathways. Several studies reported that the abnormal expression and activation of ERBB4 could lead to human cancers (Hynes and Lane, 2005; Qiu et al., 2008; Segers et al., 2020) and the loss of function due to mutations also associated with autosomal-dominant ALS (Takahashi et al., 2013). Even the TANK-binding kinase 1 (TBK1) a serine/threonine kinase, is also associated with both ALS and human cancers. It is known for its involvement in the regulation of innate immunity and autophagy through interaction with their proteins (Pottier et al., 2015). It phosphorylates p62/SQSTM1 and optineurin (OPTN) to stimulate its binding to cargo proteins and to efficiently bring them to autophagosomes for degradation (Matsumoto et al., 2015). The inhibition of TBK1 activity resulted in dendritic swellings, abnormally shaped astrocytes, cargos, and p62-and ubiquitin-positive aggregates in the cerebellum (Duan et al., 2019). Whereas, its impaired function causes suppression of cargo proteins clearance by autophagy, and contributes to the ALS (Chang et al., 2020). These mechanisms may act alone or in combination with other affected processes, therapeutically stimulating the kinase function of TBK1 may be beneficial.

The GSK3B is also a serine/threonine kinase, involved in the initiation of dynein-dependent axonal transport (Duan et al., 2019). Its activation is reported in ALS-associated disruptions in the ER/mitochondrial communication (Stoica et al., 2016), which may also moderate axonal transport indirectly *via* disrupting the ER-mitochondrial interactions. The GSK3B also phosphorylates TDP-43, while the knockout of the GSK3B gene protected against TDP-43 induced toxicity (Sreedharan et al., 2008). Although extensive research identified a direct and indirect involvement of GSK3B in ALS pathology, the real therapeutic potential in ALS patients is not yet clear. Furthermore, The JAK3 tyrosine kinase is mainly associated with the regulation of gene expression. The dysregulation of the JAK–STAT pathway occurs in inflammation and neurodegenerative disease, such as ALS (Nicolas et al., 2013). The constitutive initiation of the JAK–STAT signaling is a characteristic feature of several hematological neoplasms (Walters et al., 2006). On the other hand, the protein tyrosine kinase 2 (PTK2) also known as focal adhesion kinase (FAK), is involved in several cellular adhesion and spreading processes. The pathological role of PTK2 was reported in several advanced-stage solid cancers, recently it is identified in ALS neurodegenerative disease also (Sulzmaier et al., 2014). The leucine-rich repeat kinase 2 (LRRK2) is a large, extensively expressed, multi-domain protein, involved in several functions (Marín, 2008). The LRRK2 pathogenic mutations as well as overexpression, enhance its kinase activity and lead to cause Parkinson’s disease (Tolosa et al., 2020). While the decrease in the expression of LRRK2 has been reported to cause lung adenocarcinoma (LUAD). In patients, reduced LRRK2 was significantly associated with ongoing smoking and worse survival, as well as signatures of less differentiated LUAD, altered surfactant metabolism, and immunosuppression (Lebovitz et al., 2021). The LRRK2 is also involved in the tumorigenesis and progression of clear cell renal cell carcinoma (Yang et al., 2021). The ROCK2 (Rho-associated kinase) is a serine/threonine kinase, involved in various cellular activities such as cell adhesion and motility, actin cytoskeleton organization, smooth muscle cell contraction, remodeling of the extracellular matrix, proliferation, and apoptosis. Moreover, Rock signaling can affect differently in cellular function, depending on their regulation, subcellular localization, and other environmental factors (Hartmann et al., 2015).

### Identification of miRNAs and drug repurposing

Further, we have mapped miRNAs targeting kinases and identified 5 miRNAs including hsa-miR-16-5p, hsa-miR-124-3p, hsa-miR-27a-3p, hsa-miR-1-3p, and hsa-miR-34a-5p as the most interacting miRNAs. These miRNAs showed interaction with a maximum of 29 kinases from a total of 36 kinases. One of the identified miRNA, hsa-miR-27a-3p, is an important stimulator of adipogenesis, where it becomes downregulated during the adipogenic differentiation of Simpson-Golabi-Behmel syndrome cells, human multipotent adipose-derived cells, human primary adipose-derived stromal cells (Wu et al., 2021). The hsa-miR-27a-3p showed disruption in adipogenesis *via* inhibiting peroxisome proliferator-activated receptor γ (Wu et al., 2021). Another miRNA, the hsa-miR-1-3p has been involved in several biological functions. Its downregulation causes stimulation of proliferation and invasion in many cancers, including oral squamous cell carcinoma, colorectal carcinoma, prostate cancer, bladder cancer, and lung cancer (Zhang et al., 2019). Even, in liver injury, the hsa-miR-1-3p is upregulated and functions as a biomarker for hepatocellular injury (Kagawa et al., 2018). On the other hand, the hsa-miR-34a-5p miRNA are from the miRNA-34 family, with potential therapeutic properties. Its expression is associated with the survival of patients in colorectal cancers and is considered a marker of prognosis in earlier cancer stages (Hasakova et al., 2019). It is also reported that the overexpression of hsa-miR-34a-5p inhibits the growth of drug resistance tumors (Deng et al., 2021). Further, we identified the hsa-miR-124-3p, which significantly downregulates the plectin (PLEC) protein which connects junctions with the cytoskeleton components (Deng et al., 2021). In lung cancer, it showed the downregulation of other cellular cytoskeleton proteins including beta-1, vimentin, talin 1, cadherin 2 or N-cadherin, IQ motif containing GTPase activating protein1, and junctional adhesion molecule A (JAMA or F11R or JAM1) resulting in remodeling of cytoskeletons that causes interruption of cell–cell junctions (Deng et al., 2021). Moreover, miR-124-3p also decreases the cell adhesion capacity by directly inhibiting the formation of focal adhesion plaques. In breast cancer, it controls the NF-κB pathway by inhibiting AKT3, and moderated migration, proliferation, invasion, and inducing apoptosis (Wang et al., 2021).

Moreover, we have also identified three potential drugs including the PF-00562271, Cenisertib, and Vandetanib, targeting the kinases. These drugs result in high interaction with about a total of 18 kinases out of 36. The PF-00562271 is one of the potent dual and reversible ATP-competitive inhibitors of FAK and PYK2 (Roberts et al., 2008) and has shown interaction with about 13 kinases out of 36 kinases. The FAK is a cytoplasmic protein tyrosine kinase, that showed upregulation in several cancers such as breast, thyroid, liver, esophageal, colon, prostate, head, and neck (Stokes et al., 2011). In pancreatic ductal adenocarcinoma (PDA), the elevated expression of FAK showed a correlation with poor survival rates (Miyazaki et al., 2003; Itoh et al., 2004) and tumor size (Furuyama et al., 2006). The PF-00562271 inhibits phosphorylation of FAK in epidermal squamous cell carcinoma (Roberts et al., 2008) and Ewing sarcoma cell lines (Crompton et al., 2013), which results in the suppression of downstream pathways. Moreover, it is also involved in impairment of T cell proliferation, adhesion to intercellular adhesion molecule-1 (ICAM-1), and interactions with antigen-presenting cells (Wiemer et al., 2013). In preclinical studies, the combination of PF-00562271 with Sunitinib (multi-targeted RTK inhibitor (RTKi)), results in the suppression of proliferation and angiogenesis in the liver and epithelial ovarian cancers (Bagi et al., 2009; Stone et al., 2014). Furthermore, the PF-00562271 phase1 clinical trial (NCT00666926) was also conducted to evaluate the safety profile (Infante et al., 2012). Another identified drug target, CENISERTIB is a highly potent inhibitor of Aurora kinases. The aurora kinases are serine/threonine kinases, involved in the cell cycle *via* stimulation of mitotic spindles, while its overexpression is associated with several human cancers and its suppression by Cenisertib disrupt cell division and induce apoptosis (Mou et al., 2021). Cenisertib inhibits the kinase activity of AKT as well as FLT3, VEGFR2, LYN, BTK, and KIT and promotes growth inhibition, cell cycle arrest, and apoptosis in many cancer cell lines (McLaughlin et al., 2010). The phase-I trial (NCT00391521) of cenisertib in advance solid tumors and hematological malignancies reported early evidence of tolerance in patients with leukemia (Sonet et al., 2008). The other drug, Vandetanib is a multifunctional tyrosine kinase inhibitor (Commander et al., 2011). It acts as an orally active antagonist of EGFR/HER1, VEGFR-2 and is rearranged during transfection (RET) kinase (Commander et al., 2011). The PubChem data showed several studies reporting clinical trials of Vandetanib for many human diseases and around 23 of them completed either phase I or phase I/II trials (Supplementary Table S8). The Vandetanib results in a promising candidate for the treatment of progressive medullary thyroid cancer, and biliary tract cancers (Bianco et al., 2006; Yoshikawa et al., 2009), while in metastatic pancreatic cancer it reduced primary pancreatic tumor growth and decrease lymph node. Whereas, for liver metastasis vandetanib showed inhibition of tumor growth with gemcitabine in combination (Conrad et al., 2007).

We also performed survival analysis and found 2 two kinases that are associated with survival in LGG patients.

## Conclusion

In this study, we have used a network-based system biology approach to investigate the kinase-based molecular interplay between ALS and other human diseases including cancer. We constructed the disease-kinase interactome that demonstrates the significant involvement of kinases in several human diseases including ALS, schizophrenia, bipolar disorder, depression, and different cancers. Here, from the PPI network, the resulting hub genes including AKT1, GNB2L1, SRC, FYN, and mTOR show a high degree of interactions between the kinases. Moreover, we also identified 28 kinases including hub genes, that are involved in ALS as well as various human cancers.

Owing to its pleiotropic nature, kinases have been considered as the potential target for human diseases. We further, identified 5 miRNAs and 3 potential drug candidates by drug repurposing approach. We believe our results will help to understand the molecular interplay between ALS and other diseases by targeting kinases and this understanding of the association between ALS and other human diseases may provide a new insight for future therapeutic strategies.

## Data availability statement

The original contributions presented in the study are included in the article/Supplementary material, further inquiries can be directed to the corresponding author.

## Author contributions

FK: data curation, visualization, investigation. ShH: data curation, software. AH: data curation, visualization. AM and HT: investigation. DM: writing-original draft preparation. StH and BA: reviewing and editing. VK: conceptualization, supervision, writing, reviewing and editing. All authors contributed to the article and approved the submitted version.

## Funding

This research work was funded by the Institutional Fund projects under grant no. (IFPIP:1867-141-1443). Therefore, the authors gratefully acknowledge technical and financial support from the Ministry of Education and King Abdulaziz University, Deanship of Scientific Research, Jeddah, Saudi Arabia.

## Conflict of interest

The authors declare that the research was conducted in the absence of any commercial or financial relationships that could be construed as a potential conflict of interest.

## Publisher’s note

All claims expressed in this article are solely those of the authors and do not necessarily represent those of their affiliated organizations, or those of the publisher, the editors and the reviewers. Any product that may be evaluated in this article, or claim that may be made by its manufacturer, is not guaranteed or endorsed by the publisher.

## Supplementary material

The Supplementary material for this article can be found online at: https://www.frontiersin.org/articles/10.3389/fnmol.2022.1023286/full#supplementary-material
