# Supplementary material for: Network-based approach for targeting human kinases commonly associated with amyotrophic lateral sclerosis and cancer
Source: Front Mol Neurosci. 2022 Dec 16;15:1023286. doi: 10.3389/fnmol.2022.1023286 (PMC9802580; doi:10.3389/fnmol.2022.1023286)
Supplement: Supplementary file 2 [file Image_1.pdf]

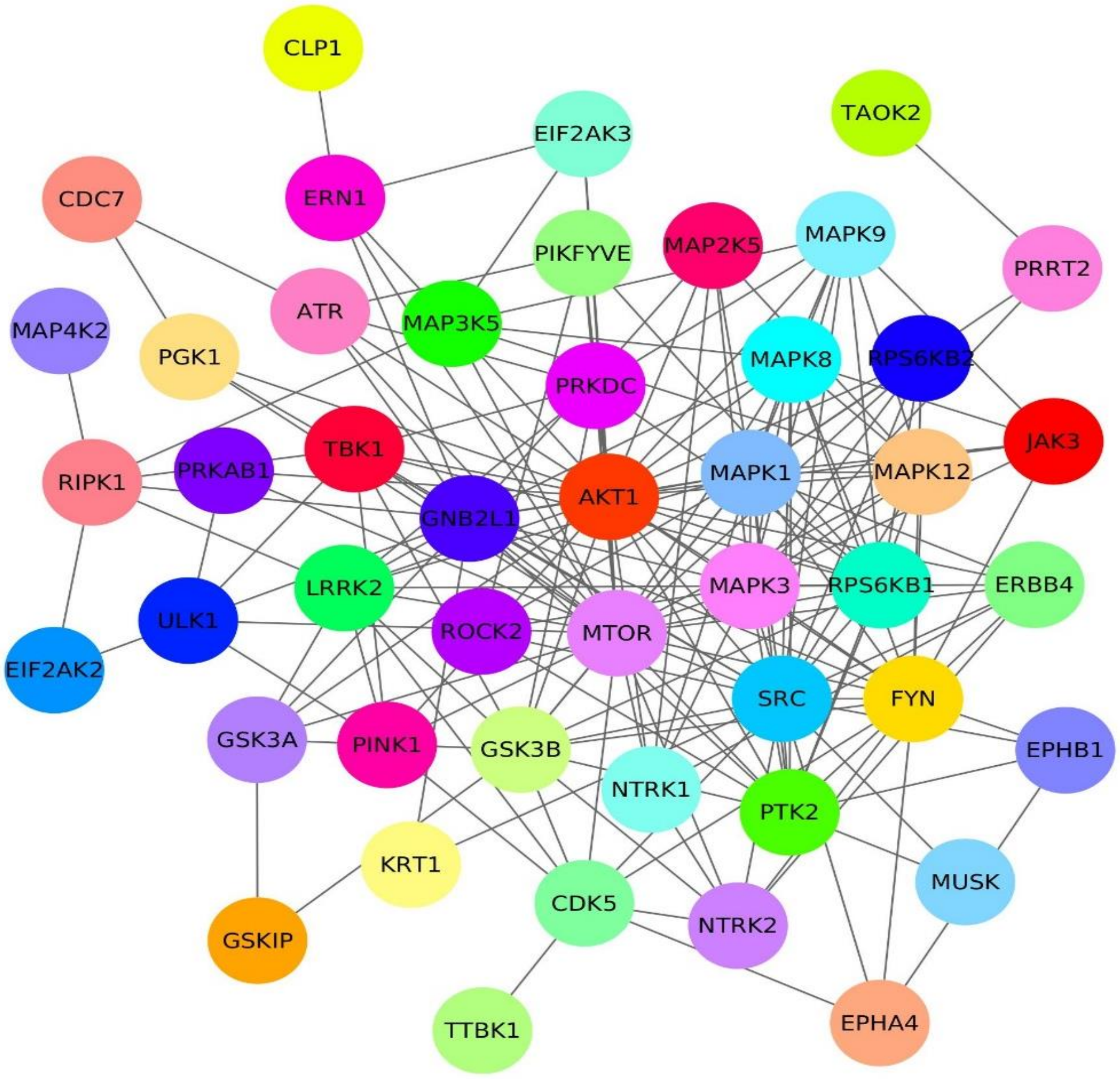

Supplementary Figure S1. Protein-Protein Interaction network of the identified ALS-associated kinases
